# The Malleted Foil Filling

**Published:** 1918-09

**Authors:** J. M. Prime


### THE MALLETED FOIL FILLING

BY J. M. PRIME, D.D.S.

What do we seek to accomplish in the operation of filling a tooth? Is not the primary object to stop the progress of decay toward the dental pulp, and save its life? To preserve it in its health? To immunize the tooth surfaces? To restore the tooth to its original form and function, and to protect the investing tissues? If this then is our duty, I ask what material has time proven to be first in accomplishing these results? With the rubber dam in place, the cavity is prepared under dry conditions, with less pain, and we know we secure a superior cavity. We know when we fill that cavity with gold foil, fresh from the flame, we are using a sterile material, and we have had demonstrated for forty years the immunity of those fillings by men who have gone before us. When properly performed, these operations have stood the test of time, first in order of all the filling materials. I plead with you, do not let the meteor going through the heavens hide from view the stars of the first magnitude. Do not let the inlay draw your attention away from the technic of the gold foil, because in the average number of cases I believe we know and can prove that gold foil can be not only better adapted to those cavity walls, but quicker. It is foolishness to discuss which is the quicker.

That is not what we are after. Which is the better? What is the material with which we may use to our great advantage the elasticity of the dentin, and place in the cavity a filling which the dentin will grip continuously and hold there secure against moisture and bacterial invasion? Only one material—gold foil. Somebody said it was so difficult to use. I challenge that statement. It is difficult because we have not studied it; it is difficult because we do not comprehend the facts entering into the operation. Five or six things are necessary, we are told, in securing a good inlay: temperature, change in volume of materials used, etc., etc. The operator in gold foil may secure absolute adaptation of the material to sterile cavity walls and eliminate all chances, in one operation. Where gold foil is indicated let us use it honestly, carefully and scientifically. It will then demonstrate its wonderful possibilities.

If you were placed upon the witness stand to testify which were the better filling, I think you would be asked how long you have used gold inlays. The reply might be, six or eight years. You would be asked how long you had used gold foil, or how long has gold foil been used by the older successful operators? The reply would be, forty or fifty years. Do you think it fair to try to establish facts on eight or ten years' experience for or against the inlay? We have not sufficient data to determine what the inlay may do. What we do have indicates to us that it is far inferior to the gold foil operation. And in view of the wonderful value of dental pulp, as has been demonstrated to us so fully recently, let us plead for the more careful and more conscientious use of a filling material that will stop permanently the progress of decay toward the valuable dental pulp. We are learning God made no mistake in placing the dental pulp in the tooth, and when the dentist substitutes a filling he gets into trouble. You say the pulp is not necessary in a tooth, and wonder why it is there. The dental pulp has a function and its preservation is necessary. Let us employ those principles in operative procedure that will best protect the dental pulp and best immunize the surfaces of the tooth, restore its form and best protect the investing tissues. Gold foil properly manipulated and properly handled, possesses superior qualities over all the others. The compressibility of the dentin: the separating of the teeth as you place the filling, allowing of a better restoration of the contact point and form of the tooth than when wax (for the gold inlay) is used. "Better?"—you say. Yes, better! When the patient bites into the wax to secure the occlusion, is an anatomical occlusion secured? No, you have simply registered *one* position of the mandible. A correct occlusion is secured only with *a working bite*, that is the unconscious biting is shown on the filling and by touching these worn facets with a stone from time to time a very nearly perfect articulation is secured.—*Journal N. D. A.*, August, 1918.

